# Supplementary material for: Resurgent threat in a changing climate: A 20-year bibliometric analysis of global Chikungunya research evolution and pandemic preparedness
Source: PLoS Negl Trop Dis. 2026 Feb 23;20(2):e0013959. doi: 10.1371/journal.pntd.0013959 (PMC12945310; doi:10.1371/journal.pntd.0013959)
Supplement: Attachment — Submitted filename: PNTD-D-25-01348 MD Comments.docx [file pntd.0013959.s011.docx]

**Reviewer Comments**

The manuscript by Xiaoyi *et al*., titled *Resurgent Threat in a Changing Climate: A 20-Year Bibliometric Analysis of Global Chikungunya Research Evolution and Pandemic Preparedness* is a comprehensive bibliometric analysis of global chikungunya virus (CHIKV) research over the past two decades, with a focus on thematic evolution, collaboration networks, and alignment with pandemic preparedness. The study is relevant, especially in light of climate-driven arboviral resurgence. The methodology is robust, and the findings offer valuable insights into research priorities, gaps, and future directions. The text is well-organized and data-rich, though several areas could benefit from clarification and refinement. Below are some comments to improve clarity and contextual depth, to make it suitable for publication.

**Major Comments**

1. **Methodological**

The authors should provide a clearer description of the inclusion/exclusion criteria, particularly regarding how duplicates and non-English records were handled (especially leads to study limitations) when this or the exclusion of generic terms like "review" and "study".

Please also clarify whether the keyword normalization process was automated or manual.

2. **Publication Volume**

The authors should elaborate more on what they called Foundational Phase (2004-2013) while Chikungunya was discovered in 1952 and 2004 coincides with Indian ocean island La Reunion important outbreak…

3. **Interpretation of Collaboration Networks**

Concerning the analysis of institutional and national collaboration the authors could better contextualize the implications of asymmetrical partnerships. For example, what are the consequences of limited equity in collaborations for endemic regions?

The authors should discuss absence of African countries in the national contributions.

How do authors explain absence of African-based institutions from international collaborations?

4. **Thematic Evolution and Keyword Analysis**

The authors should elaborate more on the rationale for selecting the top 50 keywords and the threshold for co-occurrence.

The authors interpreted the decline in “emergency response” and “crisis management” keywords as a shift toward proactive preparedness. Could this also reflect a publication bias or changing terminology?

5. **Climate Change**

Through this manuscript, the authors link climate change to CHIKV resurgence. However, the discussion could be strengthened by referencing specific climate modeling studies or projections that support the observed trends.

6. **Vaccine Development**

With the emergence of vaccine candidates like VLA1553 and IXCHIQ mentioned by the authors, it would be helpful to include a summary of their current regulatory status and deployment plans, especially in endemic regions.

Also, authors could discuss potential barriers to vaccine uptake as it was the case with Covid-19 vaccines.

**Figures 6–8** are dense and may benefit from simplification or clearer legends.
