# Supplementary material for: Resurgent threat in a changing climate: A 20-year bibliometric analysis of global Chikungunya research evolution and pandemic preparedness
Source: PLoS Negl Trop Dis. 2026 Feb 23;20(2):e0013959. doi: 10.1371/journal.pntd.0013959 (PMC12945310; doi:10.1371/journal.pntd.0013959)
Supplement: Attachment — Submitted filename: response to reviewers-9.17.docx [file pntd.0013959.s014.docx]

# Response to Reviewers

Dear editors and Reviewers,

We sincerely appreciate the constructive comments and valuable suggestions from the editorial team and all reviewers. These insightful comments have greatly helped us improve the quality and rigor of our manuscript. We have carefully revised the manuscript according to your review comments and highlighted the changes in the “Revised Manuscript with Track Changes”. Below is our detailed, point-by-point response to the reviewers’ comments.

## Response to Reviewer #1

### Comment 1

*“Introduction. There are several mentions of climate change, but no references are provided or really any effort to link climate change to the review, which is focused on the publication trends in CHIKV research. Revise the manuscript to include more introduction to the climate change link to CHIKV and describe how this is relevant to the literature review. This may be solved by moving the text from the discussion on page 28 beginning “This aligns with the epidemiological trajectory…” and ending with “… perfect ecological storm for the virus’s re-establishment.” Alternatively, I suggest removing the climate change discussion. It is not strictly necessary to the central messages of the manuscript.”*

**Response**: We are grateful to you for this valuable suggestion, which has helped us strengthen both the scientific rigor and the clarity of our work. To strengthen the link between climate change and CHIKV research (the core focus of our bibliometric analysis), we have moved the text from the Discussion section (original Page 28: “This aligns with the epidemiological trajectory…” to “… perfect ecological storm for the virus’s re-establishment”) to the Introduction section (revised manuscript Page 6–7).

Comment 2

*“Section 2.4. For transparency and reproducibility, I suggest that the merged terms, etc be described in a supplementary table.”*

**Response**: We have addressed this by adding a new supplementary table titled “Supplementary Merged Keywords”. This file comprehensively covers 654 unique keywords relevant to our study focus (arboviruses, mosquito-borne diseases, vector biology, and related research domains). Each entry in the file follows a clear two-column structure:

“label”: The original, non-standardized keyword extracted from the literature (e.g., “aedes mosquitoes”, “chikv”); “replace by”: The unified, standardized term adopted in our analysis to ensure consistency (e.g., “aedes mosquito”, “chikungunya virus”).

### Comment 3

*“There is a bias in the data, in that it only discusses English language articles but displays them globally. This is the biggest issue with this manuscript. This is acknowledged in the limitations section, but should be acknowledged in the methods.”*

**Response:** Thank you very much for your valuable comment regarding the potential bias in the data—specifically the initial focus on English-language articles while presenting global patterns. In response to this concern, we have taken three key actions:

Supplemented non-English literature: We have added Additional File: nonenglish publications 151.txt to the manuscript. This supplementary material systematically compiles non-English language studies relevant to our research topic, including publications in French, Spanish, Portuguese, German, and Turkish. These studies cover underrepresented regions such as Latin America, Southern Europe, and West Asia, addressing the linguistic and geographical imbalance in the initial dataset.

Updated results and figures: We have thoroughly revised all result sections and corresponding figures to integrate findings from these non-English publications. After incorporating these additional 151 non-English publications and re-running the analyses, the observed patterns remain essentially consistent with those reported in the original submission, and the main conclusions of our study are not altered.

### Additionally, we have explicitly acknowledged the inclusion of non-English publications and the updated data integration process in the Methods section (see revised Section 2.2 Screening and Inclusion Criteria, revised manuscript Page 8), whereas this was previously only noted in the Limitations. This revision ensures full transparency about the scope of our data sources from the outset.

### Furthermore, we clarified that English continues to be the primary language for international scientific discourse.The majority of high-impact journals and databases (including Web of Science) are English-language based, and researchers worldwide often prioritize English for broader dissemination. Therefore, while our initial dataset was English-focused, it still captures a substantial and representative portion of global CHIKV research output.

### Comment 4

*“It would be useful to reiterate the English language bias in section 3.2.1, as nodes of articles from Brazil, India, and China may have more non-English publications, where national contributions are expressed. Note that English is the language of the UK and US. The authors draw conclusions on international relationships, without more clearly acknowledging the bias.”*

**Response**: We have revised Section 3.2.1 “National Contributions” (revised manuscript Page 15) to explicitly reiterate the English-language bias when interpreting regional contributions:

“It is important to note that this distribution may be influenced by the fact that most relevant research is published in English: Brazil (with significant Portuguese-language research), India (regional languages), and China (Mandarin) likely have additional non-English publications that are not captured here, which may underestimate their actual research output and could affect interpretations of international collaboration patterns.”

### Comment 5

*“The statements in the cluster specific dynamics are affected by the English only bias. While the USA cluster has diverse global partnerships, it is unclear if Brazil has better international partnerships in Portuguese or Spanish language. This also applies to the Other Countries, including the Asia-Pacific. It is not surprising to me that Chinese institutions are publishing less in English language journals than US institutions. This needs to be included in the discussion.”*

**Response**: We have integrated this point into the Discussion section (Section 4.3, revised manuscript Page 33-34):

“The English-language restriction of our data also impacts interpretations of cluster-specific collaboration dynamics. For example, while the USA cluster shows diverse global partnerships in English-language publications, Brazil may have stronger regional collaborations documented in Portuguese or Spanish (e.g., with Latin American countries) that are not captured here. Similarly, Chinese institutions—though active in CHIKV research—publish a portion of their work in Mandarin-language journals, leading to potential underestimation of their collaboration frequency in our English-focused analysis. This bias should be considered when generalizing our findings on international research networks.”

However, it is important to note that English is the language most commonly used for global scientific research. Most international collaborations—even those involving non-English-speaking countries—are often documented in English-language publications to reach a wider audience. Therefore, while our analysis may undercount certain regional partnerships, it still reflects the dominant patterns of international collaboration in the global scientific arena.

### Comment 6

*“At the bottom of page 33, there is a typo. There needs to be a space after the period: “implications.Mitigating””*

**Response**: We have corrected this typo in the revised manuscript (revised manuscript Page 36). The sentence now reads: “implications. Mitigating”.

### Comment 7

*“In the conclusions, when discussing equitable partnerships, perhaps language should also be discussed.”*

**Response**: We have revised the Conclusions section (revised manuscript Page 38) to include language equity as a consideration for equitable research partnerships:

“To foster equitable global CHIKV research partnerships, future initiatives should address not only resource gaps but also language barriers—for example, supporting the translation of key research findings into non-English languages. This would ensure more inclusive representation of regional research contributions and strengthen collaborative pandemic preparedness.”

## Response to Reviewer #2

### Comment 1

*“data analysis and visualisation has not been discussed.”*

**Response**: We would like to thank you for pointing this issue. We have added a new subsection titled “2.5 Data Analysis and Visualization” in the Methods section (revised manuscript Page 11) to detailedly describe the analytical approaches and tools:

“2.5 Data Analysis and Visualization

Bibliometric data were analyzed using two complementary tools: VOSviewer (version 1.6.20) and R (version 4.4.3, with packages ‘circlize’, ‘ggmap’, ‘RColorBrewer’, ‘tidyverse’, ‘readxl’, ‘ggalluvial’, ‘viridis’, ‘dplyr’, ‘ggplot2’ and ‘writexl’.

Publication trend analysis: Annual publication counts and citation metrics were calculated using ‘circlize’, ‘ggmap’, ‘RColorBrewer’, ‘tidyverse’, ‘readxl’ to characterize temporal trends in CHIKV research.

Collaboration network analysis: Institutional and national collaboration networks were visualized in VOSviewer using the “co-authorship” module, with node size representing publication volume and edge thickness representing collaboration frequency.

Thematic evolution analysis: Keyword co-occurrence networks (top 51 keywords by frequencies ≥ 50) were constructed to identify thematic clusters (e.g., “Epidemiology”, “Vaccine Development”) and their temporal changes ("2016-2017" vs "2018-2019" vs "2020-2021" vs "2022-2023" vs "2024-2025") using ‘tidyverse’, ‘readxl’, ‘ggalluvial’, ‘viridis’, ‘dplyr’, ‘ggplot2’ and ‘writexl’.

Visualization quality control: All figures were optimized for clarity (e.g., adjusting node spacing in networks, standardizing color scales for thematic clusters).”

### Comment 2

*“Major: The results and discussions are disjointed. Since this is a bibiometric analysis, I anticipate the discussion will highlight the findings, significance and limitations from this analysis, rather than discussing the epidemiology (section 4.1), vaccine development (4.2) etc. There are many sections of over-interpretation of data. One example is in 4.3 Public Health Strategies and Their Shortcomings, the authors discussed the decline in “Emergency Response and Crisis Management” keywords (16.2% to 7.2% from 2016-2017 to 2024-2025) within public health research suggests a problematic transition away from reactive capacity, despite the ongoing threat. There are many factors that can contribute to the decline in certain keywords, and as there is no objective evaluation (qualitative/quantitative measure) presented here.”*

**Response**: We sincerely thank you for this insightful comment. We agree that the Discussion section should be tightly focused on interpreting the bibliometric findings, their implications, and limitations, rather than providing a general narrative review of CHIKV epidemiology or vaccine development. We have thoroughly revised the entire Discussion section (Section 1-3) to ensure it:

Explicitly links each discussion point to specific findings from our analysis (e.g., trends in publication volume, keyword evolution, collaboration patterns).

Avoids over-interpretation by acknowledging alternative explanations for observed trends (e.g., the decline in certain keywords could reflect shifting research priorities rather than a loss of capacity).

Highlights the significance and limitations of our bibliometric approach itself.

The revised discussion is now structured around the core themes emerging from our data, ensuring a cohesive narrative that flows directly from the results.

### Comment 3

*“The selection of English language publication alone in this analysis is a key limitation when assessing equitable partnerships.”*

**Response:** We sincerely thank you for raising this crucial point. We fully agree that relying solely on English-language publications introduces a significant limitation when assessing global research equity and partnership patterns, as it systematically underestimates contributions from regions where research is often published in other languages. In response to this concern, we have taken three key actions:

Supplemented non-English literature: We have added Additional File: nonenglish publications 151.txt to the manuscript. This supplementary material systematically compiles non-English language studies relevant to our research topic, including publications in French, Spanish, Portuguese, German, and Turkish. These studies cover underrepresented regions such as Latin America, Southern Europe, and West Asia, addressing the linguistic and geographical imbalance in the initial dataset.

Updated results and figures: We have thoroughly revised all result sections and corresponding figures to integrate findings from these non-English publications. After incorporating these additional 151 non-English publications and re-running the analyses, the observed patterns remain essentially consistent with those reported in the original submission, and the main conclusions of our study are not altered.

### Additionally, we have explicitly acknowledged the inclusion of non-English publications and the updated data integration process in the Methods section (see revised Section 2.2 Screening and Inclusion Criteria, revised manuscript Page 8), whereas this was previously only noted in the Limitations. This revision ensures full transparency about the scope of our data sources from the outset.

### Furthermore, we clarified that English continues to be the primary language for international scientific discourse.The majority of high-impact journals and databases (including Web of Science) are English-language based, and researchers worldwide often prioritize English for broader dissemination. Therefore, while our initial dataset was English-focused, it still captures a substantial and representative portion of global CHIKV research output.

### Comment 4

*“Minor: Page 12, under 3.2.1 National Contributions: “The United States (USA) dominated with 2,622 publications, constituting (2.9% of the total… Is this 29% rather than 2.9%?””*

**Response**: Thank you for pointing out this typographical error. We have corrected the percentage in the revised manuscript (revised manuscript Page 15) . The corrected sentence reads: *“*The United States (USA) dominated with 2,626 publications, constituting (32.3% of the total, followed by Brazil with 1,240 publications (15.3%), France with 1,067 (13.1%), and India with 1,011 (12.4%). *”*

## Response to Reviewer #3

### Comment 1

*“1.Methodological*

*The authors should provide a clearer description of the inclusion/exclusion criteria, particularly regarding how duplicates and non-English records were handled (especially leads to study limitations) when this or the exclusion of generic terms like "review" and "study".*

*Please also clarify whether the keyword normalization process was automated or manual.”*

**Response**: Thank you for raising these important methodological points. We have revised the Methods section (Section 2.2) to provide greater clarity regarding our screening process:

Duplicates: We identified and removed duplicates using a two-step process: first, automated deduplication in EndNote X20 based on DOI and title matching; second, manual verification to resolve any discrepancies (e.g., variations in author names or publication years). This process identified 6 duplicate records, which were excluded, resulting in a final analytic dataset of 8,125 unique publications. (revised manuscript Page 9)

Non-English records: Initially, only English-language publications were included, but we have now supplemented our analysis with relevant non-English publications (see Additional File 151). These have been integrated into the updated results and figures to provide a more globally representative dataset.

Exclusion of generic terms: Generic terms such as “review,” “study,” and “article” were excluded during keyword normalization to focus the analysis on substantive research themes. (revised manuscript Page 10)

Keyword normalization: The keyword normalization process was semi-automated. We first used the bibliometrix package in R to group variants algorithmically (e.g., “CHIKV” and “chikungunya virus”), followed by manual review to ensure accuracy and consistency. A full list of merged keywords is provided in Supplementary merged keywords.xlsx.

These clarifications have been incorporated into the revised manuscript to enhance transparency and reproducibility.

### Comment 2

*“2.Publication Volume*

*The authors should elaborate more on what they called Foundational Phase (2004-2013) while Chikungunya was discovered in 1952 and 2004 coincides with Indian ocean island La Reunion important outbreak….”*

**Response**: We thank you for this insightful comment. We fully agree that the historical context of chikungunya discovery and earlier outbreaks is important. The term “Foundational Phase” in our analysis refers specifically to the modern re-emergence of CHIKV, which began with the large-scale outbreak on Réunion Island and surrounding Indian Ocean islands in 2004–2005. This event marked a turning point in CHIKV research due to the unprecedented scale of the outbreak, the emergence of a new viral lineage (Indian Ocean lineage, IOL), and the virus's adaptation to Aedes albopictus, which significantly expanded its geographic potential.

Although CHIKV was first identified in 1952, research output remained relatively limited until the 2004–2005 outbreak, which triggered a sustained increase in scientific publications and global attention. To clarify this point, we have revised the manuscript in Section 3.1 (Analysis of Publication Volume, revised manuscript Page 12-13) to better contextualize the “Foundational Phase” within the broader history of CHIKV:

“(1) Foundational Phase (2004-2013) Although chikungunya virus was first identified in 1952, research activity remained limited until the large-scale re-emergence of CHIKV in the Indian Ocean islands (e.g., Réunion) in 2004–2005. This outbreak, caused by a new lineage (Indian Ocean lineage) with enhanced transmission by Aedes albopictus, marked the beginning of the modern era of CHIKV research. During this phase, annual output averaged 109 publications per year, rising from a low of 7 publications in 2005 to a peak of 230 in 2013. This period established the foundational research framework for subsequent studies but accounted for only 13.4% (n = 1,091) of the total output. ”

### Comment 3

*“3.Interpretation of Collaboration Networks*

*Concerning the analysis of institutional and national collaboration the authors could better contextualize the implications of asymmetrical partnerships. For example, what are the consequences of limited equity in collaborations for endemic regions?*

*The authors should discuss absence of African countries in the national contributions.*

*How do authors explain absence of African-based institutions from international collaborations?”*

**Response**: We sincerely thank you for raising these critical points. We agree that the implications of collaboration asymmetry—particularly for endemic regions like Africa—warrant deeper contextualization. We have revised the Discussion section (Section 4.3) to explicitly address:

The consequences of limited equity in collaborations for endemic regions; The underrepresentation of African countries in the national contribution analysis; Potential reasons for the absence of African-based institutions in international collaboration networks.

Below is the revised text added to the manuscript on revised manuscript Page 34:

“Furthermore, the scarcity of African countries among the top publishing nations—despite high disease burden—highlights a critical equity gap. This absence may stem from structural barriers such as limited research funding, language biases in international indexing, and lower visibility of regional journals. The underrepresentation of African institutions in collaboration networks risks perpetuating a knowledge imbalance, wherein local expertise, ecological insights, and implementation contexts are undervalued. This may lead to research agendas that are misaligned with regional priorities, inefficient resource allocation, and interventions that are less effective in local settings. Strengthening equitable partnerships with endemic regions in Africa and elsewhere is not only an ethical imperative but also essential for developing contextually appropriate and sustainable public health strategies.”

### Comment 4

*“4.Thematic Evolution and Keyword Analysis*

*The authors should elaborate more on the rationale for selecting the top 50 keywords and the threshold for co-occurrence.*

*The authors interpreted the decline in “emergency response” and “crisis management” keywords as a shift toward proactive preparedness. Could this also reflect a publication bias or changing terminology?”*

**Response**: Thank you for this important question regarding the methodological rationale for our keyword analysis.

In the co-occurrence analysis of author keywords using VOSviewer, we set the minimum number of occurrences of a keyword to 50. This threshold was selected to ensure that the resulting network reflects well-established, recurrent themes within the chikungunya research landscape, while maintaining clarity and interpretability of the visualized clusters. A total of 51 keywords met this threshold and were included in the final co-occurrence network.

This approach is consistent with common practices in bibliometric studies, as it helps filter out less frequent or peripheral terms, thereby highlighting the core research topics and conceptual structure of the field. The threshold of 50 occurrences strikes a balance between inclusiveness and focus, allowing us to robustly identify major thematic areas without excessive noise from low-frequency terms.

We fully agree with you that a decline in specific keywords may reflect not only a genuine shift in research focus but also potential publication biases or evolving terminology. In the revised Discussion section (Section 4.2), we now explicitly acknowledge this ambiguity and avoid over-interpretation. We state:

“Conversely, the proportional decline in "Emergency Response and Crisis Management" keywords (-8.3%) (Section 3.6.4, Figure 8A) is a critical finding. While this might suggest a desirable transition toward pre-emptive preparedness, it could also indicate a concerning de-prioritization of reactive outbreak capacity—a capability that remains essential given the virus's unpredictable emergence. Bibliometric data alone cannot distinguish between these interpretations; this decline may reflect a maturation of the field beyond initial outbreak response reports, a shift in terminology, or a genuine gap. Therefore, we interpret this trend not as a definitive measure of capacity loss, but as a signal warranting further qualitative investigation into whether research is adequately supporting frontline outbreak response needs.” (revised manuscript Page 32-33)

### Comment 5

*“5.Climate Change*

*Through this manuscript, the authors link climate change to CHIKV resurgence. However, the discussion could be strengthened by referencing specific climate modeling studies or projections that support the observed trends.”*

**Response**: We thank you for this valuable suggestion. We agree that referencing specific climate modeling studies would strengthen the discussion on the link between climate change and CHIKV transmission dynamics.

In response, we have revised the Discussion (Section 4.4) to include citations to recent climate modeling projections that support our bibliometric observations. Specifically, we now reference and state:

“Dai et al. (2025), who conducted a global assessment of current and future CHIKV transmission risk using optimized MaxEnt modeling. Their projections under various climate scenarios strongly support the expansion of climatically suitable areas for CHIKV transmission, thereby providing a mechanistic foundation for the observed research trends and the virus's potential for resurgence.” (revised manuscript Page 35-36)

### Comment 6

*“6.Vaccine Development*

*With the emergence of vaccine candidates like VLA1553 and IXCHIQ mentioned by the authors, it would be helpful to include a summary of their current regulatory status and deployment plans, especially in endemic regions.*

*Also, authors could discuss potential barriers to vaccine uptake as it was the case with Covid-19 vaccines.*

*Figures 6–8 are dense and may benefit from simplification or clearer legends. ”*

**Response**: We are grateful to you for these insightful suggestions, which have helped us significantly enhance the discussion on vaccine development.

(1)Regulatory Status and Deployment Plans: To contextualize the observed keyword emergence of 'VLA1553' and 'IXCHIQ'—a core result from our analysis—we have added a sentence summarizing their current status:

"This trend aligns with tangible progress in the field, exemplified by the first regulatory approvals of a chikungunya vaccine (VLA1553, marketed as IXCHIQ®) by the U.S. FDA in late 2023 and other agencies thereafter, with initial deployment strategies currently targeting high-risk groups in endemic regions." (revised manuscript Page 32)

(2)Potential Barriers to Vaccine Uptake: Acknowledging your crucial point, we have added a discussion on potential challenges to vaccine implementation, drawing lessons from other vaccine campaigns:

"Despite this milestone, the path to widespread vaccine adoption may face significant barriers. Lessons from COVID-19 and other vaccine introductions highlight potential hurdles, including vaccine hesitancy, logistical challenges related to cold-chain requirements for live-attenuated vaccines, cost-effectiveness considerations in resource-limited settings, and the need for robust pharmacovigilance systems. Proactively addressing these implementation challenges is essential for ensuring equitable access and achieving successful integration of chikungunya vaccines into public health programs in endemic regions."(revised manuscript Page 32)

To address this issue as you suggested, we have revised Figures 6–8. The key modification is that we have relocated the figure legends from their original position to the side of the figures. This adjustment not only reduces visual density but also makes the legends more accessible, thereby enhancing the overall readability of the figures.

Once again, we sincerely thank the reviewers for your time and expertise, which have substantially improved our manuscript. We hope that the revised version satisfactorily addresses all concerns and is suitable for publication in PLOS Neglected Tropical Diseases. We look forward to your further review.

Sincerely,

Minyi He (Corresponding Author)

On behalf of all co-authors
