# Supplementary material for: Resurgent threat in a changing climate: A 20-year bibliometric analysis of global Chikungunya research evolution and pandemic preparedness
Source: PLoS Negl Trop Dis. 2026 Feb 23;20(2):e0013959. doi: 10.1371/journal.pntd.0013959 (PMC12945310; doi:10.1371/journal.pntd.0013959)
Supplement: Attachment — Submitted filename: PNTD-D-25-01348 R1 MD Comments.docx [file pntd.0013959.s013.docx]

Thank you for sharing the revised manuscript and the authors’ detailed responses to my comments.

I have reviewed their replies and the corresponding revisions with appreciation. Regarding **Comment 1 (Methodological clarity)**, the authors have provided a more transparent account of their inclusion/exclusion criteria, particularly in relation to duplicate handling, non-English records, and keyword normalization. The clarification of the semi-automated process using the bibliometrix package, followed by manual verification, is appropriate and enhances reproducibility. The integration of non-English publications and the rationale for excluding generic terms such as “review” and “study” are also well justified and improve the global representativeness of the dataset.

For **Comment 2 (Publication Volume and the “Foundational Phase”)**, I acknowledge the authors’ effort to contextualize the term within the modern re-emergence of CHIKV following the 2004–2005 outbreak in the Indian Ocean region. The revised section now better reflects the historical trajectory of chikungunya research and appropriately distinguishes between early identification and the surge in scientific output post-2004. The explanation regarding the emergence of the Indian Ocean lineage and its epidemiological significance is convincing.

**Comment 3 – Collaboration Networks**

I appreciate the authors’ expanded discussion on asymmetrical partnerships and the underrepresentation of African countries and institutions. The revised Section 4.3 appropriately highlights structural barriers such as limited funding, language bias, and journal visibility. The acknowledgment of potential consequences (such as misaligned research agendas and undervaluation of local expertise) is well articulated and ethically grounded. I find this revision satisfactory and aligned with the intent of my comment.

**Comment 4 – Thematic Evolution and Keyword Analysis**

The authors have clarified their rationale for selecting the top 50 keywords and the co-occurrence threshold, referencing standard bibliometric practices. Their nuanced interpretation of the decline in “emergency response” and “crisis management” keywords is commendable. By acknowledging the possibility of publication bias or evolving terminology, they avoid over-interpretation and invite further qualitative inquiry. This balanced approach strengthens the manuscript’s analytical depth.

**Comment 5 – Climate Change**

The integration of recent climate modeling studies, particularly the reference to Dai et al. (2025), adds valuable context to the discussion on CHIKV resurgence. This revision provides a mechanistic foundation for the observed bibliometric trends and enhances the scientific rigor of the climate-related analysis.

**Comment 6 – Vaccine Development**

The authors have effectively addressed both aspects of my comment. The summary of regulatory status and deployment plans for VLA1553/IXCHIQ is timely and informative. Their discussion of potential barriers to vaccine uptake (drawing lessons from COVID-19) demonstrates thoughtful engagement with implementation challenges.

The adjustment to Figures 6–8, relocating legends for improved readability, is a welcome enhancement.

Overall, I find the authors’ revisions satisfactory and believe they have addressed the concerns raised. I have no further major comments at this stage.

Best regards,

**Dr. Maurice Demanou**

Reviewer #3
