# Supplementary material for: Resurgent threat in a changing climate: A 20-year bibliometric analysis of global Chikungunya research evolution and pandemic preparedness
Source: PLoS Negl Trop Dis. 2026 Feb 23;20(2):e0013959. doi: 10.1371/journal.pntd.0013959 (PMC12945310; doi:10.1371/journal.pntd.0013959)
Supplement: Attachment — Submitted filename: response to reviewers (revision 2)1017.docx [file pntd.0013959.s015.docx]

# Response to Reviewers

Dear editors and Reviewers,

We sincerely appreciate the constructive comments and valuable suggestions from the editorial team and all reviewers. These insightful comments have greatly helped us improve the quality and rigor of our manuscript. We have carefully revised the manuscript according to your review comments and highlighted the changes in the “Revised Manuscript with Track Changes”. Below is our detailed, point-by-point response to the reviewers’ comments.

## Response to Reviewer #1

### Comment 1

*Reviewer #1: This revision is a significant improvement. The English language concerns are better addressed. However, the conclusions of translating English language into other languages. However, this conclusion seems like a one-way exchange. The authors point to the low volume of American-Chinese collaborations, but give no insight into the volume of Chikungunya publications in the Chinese literature. I suggest on Page 34, after the authors state: "Similarly, Chinese institutions—though active in CHIKV research—publish a portion of their work in Mandarin-language journals, leading to potential underestimation of their collaboration frequency in our English-focused analysis." the authors should add some additional information, such as the number of articles that found in Chinese language biomedical bibliographic databases when seached for "Chikungunya". From databases such as the Chinese Biomedical Literature Database or WANFANG database. Also, I would encourage the authors to be more ambitious in their conclusions on strengthening US-Chinese research. For example, calling for joint publication of articles in both Chinese and English language.*

**Response**: Thank you for your insightful comments and for underscoring the value of non-English publications and US–China research collaboration. Accordingly, we conducted a comprehensive supplementary search in two leading Chinese bibliographic databases: the Chinese Biomedical Literature Database (SinoMed) and the WANFANG Database. The search was performed using the precise Chinese translation of "chikungunya" ("基孔肯雅热"), which identified 289 records in SinoMed and 286 in WANFANG. After a careful deduplication process, we consolidated a final collection of 350 unique Chinese-language publications on CHIKV. We have added a supplementary table (Supplementary Chinese publications 350.xlsx) to show the results and integrated the findings into the manuscript (Page 34). Please find below the revised section of our manuscript.

Some Chinese institutions published their work in Mandarin-language journals, which may not be fully captured by our English-focused analysis and could thus lead to an underestimation of their collaborative efforts. To quantify this potential gap, we performed an additional search in two major Chinese bibliographic databases (Chinese Biomedical Literature Database (SinoMed) and WANFANG Database) using the Chinese term for chikungunya ("基孔肯雅热"). This search identified 350 unique Chinese-language publications on CHIKV (Supplementary chinese publications 350.xlsx). Among the 350 publications analyzed, all Chinese-language studies were published without co-authors from the United States or other countries. This finding aligns with trends in the Web of Science database, indicating that international collaboration in chikungunya research remains relatively limited in China. In addition to the potential underrepresentation of Chinese-language publications in our analysis, this limited collaboration is also due to the historically low incidence of the disease in China. This has led to a relatively weak domestic research base and thus limited motivation or infrastructure for extensive international cooperation. However, as chikungunya continues to spread globally, China also faces growing public health risks from the disease. In light of this, research needs in China are expected to rise significantly. Strengthening collaboration with the United States and other countries in this field could greatly enhance global efforts to prevent and control chikungunya. Transforming this imbalanced architecture into a robust and equitable global network will require systematic efforts to integrate non-English research into the global knowledge commons. Key steps include supporting the translation and indexing of key findings from regional journals and incorporating databases such as SciELO and CNKI into systematic reviews and bibliometric analyses.

**Comment 2**

*“There is a typo on Page 42 where it says "preparedness.Third" there should be a space after the period.”*

**Response**: Thank you for your careful reading and for pointing out this typographical error. We sincerely appreciate you catching this oversight.

We have now corrected this by inserting a space after the period. The revised sentence reads:

"...preparedness. Third, critical gaps persist..."

This error has been corrected in the revised manuscript (on page 38).

### Comment 3

*“Much improved article, but I believe that English language Chikungunya research community would benefit from a more ambitious conclusion section, where the authors propose how to strengthen the global network, particularly how English speaking regions can engage with non-English speaking regions, with particular emphasis on China, since the authors are from Chinese institutions.”*

**Response:** Thank you for your continued guidance in strengthening our manuscript. As it relates to the point raised in Comment 1, we have applied the same approach to address it. This included a supplementary literature search in Chinese databases, as detailed in our previous response. The manuscript has been revised to incorporate these findings.

## Response to Reviewer #4

### Comment 1

*“Page 35, under 4.4 Climate Change and Urbanization: Catalysts for CHIKV Re-emergence*

*What does "systemic health" mean? Dose it refer to the overall well-being and functional integrity of the entire body, ecology, or other aspects? Please provide an explanation.”*

**Response**: We sincerely thank you for this insightful comment. The term “systemic health implications” is used to describe the multi-level and interconnected impacts of climate change and urbanization on health systems, population health, and societal resilience—extending beyond direct viral transmission. Below, we clarify the concept based on the evidence presented in our manuscript:

1.Broadening Geographic Scope and Health System Preparedness:

Climate change facilitates the expansion of Aedes mosquito habitats into temperate regions, as documented in Europe and North America. This shift introduces CHIKV into new populations lacking immunity, thereby straining local health systems unprepared for arboviral outbreaks. This represents a systemic health challenge, as it requires integrated surveillance, diagnostic capacity, and clinical management across regions.

2.Long-Term Health Burdens and Health System Resources:

CHIKV infection often leads to chronic arthralgia, which imposes sustained demand on healthcare services. Our keyword analysis (Section 3.6.4) shows a rising focus on “environmental health” and “global eco-health”, reflecting the recognition that climate-related outbreaks exacerbate long-term morbidity and necessitate coordinated care across clinical and public health domains.

3.Interplay with Social and Environmental Determinants:

Unplanned urbanization amplifies exposure risks through high population density, inadequate water management, and poor sanitation. These factors interact with social inequities—such as limited access to healthcare—creating systemic vulnerabilities that propagate across communities and generations.

4.Evidence from Predictive Modeling:

Our citation of Dai et al. (2025) reinforces that climate scenarios project continued expansion of CHIKV-suitable areas. This supports our argument that climate-driven transmission is not an isolated event but part of a systemic transition, affecting ecological, infrastructural, and health outcomes collectively.

To avoid ambiguity, we have revised the relevant sentence in Section 4.4 to explicitly state:

“…reflecting intensified scientific focus on climate-driven vector expansion and its systemic health implications, implications spanning health system capacity, long-term patient care, and social equity.”

### Comment 2

*“2.The manuscript needs to be edited by a native English speaker before it can be considered acceptable for publication.”*

**Response**: We sincerely thank the reviewer for this valuable comment. We completely agree that high-quality language editing is essential for the clarity and professionalism of the manuscript.

In response, we have engaged a professional English language editing service to thoroughly polish the entire text. The service was provided by Springer Nature Author Services, a recognized leader in academic editing. The edited manuscript has been carefully reviewed and incorporated, ensuring that the language now meets the high standards required for international publication. The certificate of language editing has been uploaded as an attachment.

Once again, we sincerely thank the reviewers for your time and expertise, which have substantially improved our manuscript. We hope that the revised version satisfactorily addresses all concerns and is suitable for publication in PLOS Neglected Tropical Diseases. We look forward to your further review.

Sincerely,

Minyi He (Corresponding Author)

On behalf of all co-authors
