# Supplementary material for: Resurgent threat in a changing climate: A 20-year bibliometric analysis of global Chikungunya research evolution and pandemic preparedness
Source: PLoS Negl Trop Dis. 2026 Feb 23;20(2):e0013959. doi: 10.1371/journal.pntd.0013959 (PMC12945310; doi:10.1371/journal.pntd.0013959)
Supplement: Attachment — Submitted filename: response to reviewers (revision 3).docx [file pntd.0013959.s016.docx]

# Response to Reviewers

Dear editors and Reviewers,

We sincerely appreciate the constructive comments and valuable suggestions from the editorial team and all reviewers. These insightful comments have greatly helped us improve the quality and rigor of our manuscript. We have carefully revised the manuscript according to your review comments and highlighted the changes in the “Revised Manuscript with Track Changes”. Below is our detailed, point-by-point response to the reviewers’ comments.

## Response to Reviewer #1

### Comment

*I did notice three unusual word usages. On page 8, "coword" should be "co-word" and "biclustering" should be "bi clustering" or "bi-clustering" (I defer to PLOS editoral staff on the correct word usage). On page 21 "Cocitation" should be "Co-citation".*

**Response**: Thank you for your careful reading and helpful suggestions regarding the terminology in the manuscript.

We have revised the text according to your recommendations: On page 8, “coword” has been changed to “co-word,” and “biclustering” has been amended to “bi-clustering.” On page 21, “Cocitation” has been revised to “Co-citation.”

All changes have been incorporated into the revised manuscript.

## Response to Reviewer #4

### Comment 1

*Reviewer #4: 1. Justify the criterion of " Justify the criterion of "minimum 50 occurrences" (e.g., whether sensitivity verification was performed)." (e.g., whether sensitivity verification was performed).*

**Response**: Thank you for your valuable comment regarding the keyword co-occurrence analysis threshold. We appreciate the opportunity to clarify the rationale behind setting the minimum occurrence threshold at 50 and to confirm that sensitivity verification was conducted.

1. Rationale for the “Minimum 50 Occurrences” Threshold:

In bibliometric co-word analysis, the selection of a keyword threshold balances network interpretability with thematic comprehensiveness. Based on our dataset of 8,125 publications, a threshold of 50 occurrences yielded 51 keywords, which we found to be optimal for the following reasons:

Thematic Representativeness: Keywords occurring ≥50 times correspond to core, consistently studied topics in chikungunya research, such as chikungunya virus, vaccine, Aedes aegypti, climate change, and epidemiology. These terms collectively reflect the main research domains: virology, vector biology, public health interventions, and environmental drivers.

Network Clarity and Stability: This threshold produced a well-structured and interpretable co-occurrence network with five coherent clusters (Core Pathogens & Diseases, Transmission Vectors, Transmission Dynamics & Epidemic Characteristics, Control Strategies & Interventions, Influencing Factors & Host Response). A lower threshold introduced noise from infrequent terms, while a higher threshold excluded meaningful themes (e.g., climate change appeared 94 times and would be retained at 50 but lost if the threshold were raised to 100).

Alignment with Bibliometric Conventions: The selection of keyword occurrence thresholds in bibliometric analysis is study-specific and varies based on the size of the dataset and analytical objectives. For instance, in an analysis of 1,308 publications on SLC26A4-related hearing loss, a threshold of 20 was employed to identify research fronts (Li et al., 2025), whereas in a larger study of 26,085 publications on osteoporosis treatment, a threshold of 100 was used to ensure clarity in keyword co-occurrence mapping (Kim & Lee, 2024). These examples illustrate that thresholds are adapted to balance comprehensiveness and focus, rather than conforming to a fixed range.

Li Y, Wen C, Yu Y, Deng L, Gao S, Huang L. Research hotspots and trends of the SLC26A4 gene-related hearing loss from the perspective of knowledge graph. Intractable Rare Dis Res. 2025 Feb 28;14(1):55-66.

Kim SJ, Lee DW. Publication Trends in Osteoporosis Treatment: A 20-Year Bibliometric Analysis. J Bone Metab. 2024;31(2):90-100.

2. Sensitivity Verification:

To ensure the robustness of our threshold selection, we performed sensitivity analyses by testing alternative thresholds:

Threshold = 40: Resulted in 66 nodes; while still representative, some less central terms (e.g., elisa, invasive specy) began to appear, slightly blurring cluster boundaries.

Threshold = 60: Reduced nodes to 41, excluding several relevant keywords (e.g., brazil, diagnostic, innate immunity) that contribute to interdisciplinary understanding.

Threshold = 50: Provided the optimal balance, retaining key interdisciplinary themes while maintaining a clean, interpretable network structure. The five thematic clusters remained stable across thresholds ≥50, confirming the robustness of the selected cutoff.

### Comment 2

2. *In Analytical Framework,A bibliometric analysis was conducted employing R 4.3.3, however, In Data Analysis and Visualization, Bibliometric data were analyzed using …… R (version 4.4.3). You should ensure the version is consistent throughout your paper and reflects the most appropriate one used for the analysis.*

**Response**: Thank you for pointing out the inconsistency in the R version number mentioned in the manuscript. We have corrected the version number to R 4.4.3 throughout the paper (including in the “Analytical Framework” and “Data Analysis and Visualization” sections) to ensure accuracy and consistency. This is the version that was used for all analyses reported in the study.

### Comment 3

*3. Relying solely on Web of Science may miss literatures from Scopus/PubMed (especially non-English regions).*

**Response**: Thank you for your valuable comment. We fully agree that relying solely on the Web of Science may limit the comprehensiveness of the literature base, especially for research from non - English regions. Although the Web of Science is a mainstream database for bibliometric studies, we have significantly expanded the Limitations section (4.5). We now explicitly discuss the potential underrepresentation of literature from other databases such as Scopus and PubMed, as well as non - English publications. Importantly, we have incorporated the results of a supplementary search in Chinese databases (SinoMed and WANFANG) as a case study to illustrate and partially characterize this gap. The findings (350 Chinese-language publications with no international co-authorship) are included in the supplementary materials and are referenced in the text.

Once again, we sincerely thank the reviewers for your time and expertise, which have substantially improved our manuscript. We hope that the revised version satisfactorily addresses all concerns and is suitable for publication in PLOS Neglected Tropical Diseases. We look forward to your further review.

Sincerely,

Minyi He (Corresponding Author)

On behalf of all co-authors
